# Knowledge domains and emerging trends of microglia research from 2002 to 2021: A bibliometric analysis and visualization study

**DOI:** 10.3389/fnagi.2022.1057214

**Published:** 2023-01-05

**Authors:** Guangjie Liu, Tianhua Li, Anming Yang, Xin Zhang, Songtao Qi, Wenfeng Feng

**Affiliations:** ^1^Department of Neurosurgery, Nanfang Hospital, Southern Medical University, Guangzhou, China; ^2^Department of Neurosurgery, Xuanwu Hospital, Capital Medical University, Beijing, China; ^3^China International Neuroscience Institute (China-INI), Beijing, China

**Keywords:** bibliometric analysis, CiteSpace, knowledge domain, microglia, VOSviewer

## Abstract

**Background:**

Microglia have been identified for a century. In this period, their ontogeny and functions have come to light thanks to the tireless efforts of scientists. However, numerous documents are being produced, making it challenging for scholars, especially those new to the field, to understand them thoroughly. Therefore, having a reliable method for quickly grasping a field is crucial.

**Methods:**

We searched and downloaded articles from the Web of Science Core Collection with “microglia” or “microglial” in the title from 2002 to 2021. Eventually, 12,813 articles were located and, using CiteSpace and VOSviewer, the fundamental data, knowledge domains, hot spots, and emerging trends, as well as the influential literature in the field of microglia research, were analyzed.

**Results:**

Following 2011, microglia publications grew significantly. The two prominent journals are Glia and J Neuroinflamm. The United States and Germany dominated the microglia study. The primary research institutions are Harvard Univ and Univ Freiburg, and the leading authors are Prinz Marco and Kettenmann Helmut. The knowledge domains of microglia include eight directions, namely neuroinflammation, lipopolysaccharide, aging, neuropathic pain, macrophages, Alzheimer’s disease, retina, and apoptosis. Microglial phenotype is the focus of research; while RNA-seq, exosome, and glycolysis are emerging topics, a microglial-specific marker is still a hard stone. We also identified 19 influential articles that contributed to the study of microglial origin (Mildner A 2007; Ginhoux F 2010), identity (Butovsky O 2014), homeostasis (Cardona AE 2006; Elmore MRP 2014); microglial function such as surveillance (Nimmerjahn A 2005), movement (Davalos D 2005; Haynes SE 2006), phagocytosis (Simard AR 2006), and synapse pruning (Wake H 2009; Paolicelli RC 2011; Schafer DP 2012; Parkhurst CN 2013); and microglial state/phenotype associated with disease (Keren-Shaul H 2017), as well as 5 review articles represented by Kettenmann H 2011.

**Conclusion:**

Using bibliometrics, we have investigated the fundamental data, knowledge structure, and dynamic evolution of microglia research over the previous 20 years. We hope this study can provide some inspiration and a reference for researchers studying microglia in neuroscience.

## Introduction

Microglia are resident macrophages in the central nervous system (Prinz et al., 2019). Since Río Hortega first discovered and described them in 1919 (Sierra et al., 2019), they have advanced quickly thanks to the advancements in molecular biology, microscope imaging technology, and single-cell sequencing technology (Bohlen et al., 2019). As macrophages derived from the yolk sac (Ginhoux et al., 2010), microglia can not only be transformed by bone marrow-derived macrophages (which can be restricted by the blood–brain barrier; Mildner et al., 2007) but also achieve self-renewal (Ajami et al., 2007) in the brain.

As a critical intracranial immune cell, it has been proven to play an essential role in the central nervous system’s development, maturation, homeostasis, and disease (Bohlen et al., 2019). In neural development and the maintenance of adult brain homeostasis, it plays an essential role in the phagocytosis of dead cells, pruning synapses, monitoring synaptic activity, rebuilding neural circuits, and exhibiting immune activity. Microglia are closely related to many diseases, such as stroke (Ma et al., 2017), Alzheimer’s disease (AD) (Boche and Gordon, 2022), Parkinson’s disease (PD) (Joers et al., 2017), depression (Yirmiya et al., 2015), schizophrenia (Xia et al., 2020), and traumatic brain injury (TBI) (Morganti-Kossmann, 2019). Microglia can change their phenotype in a disease state, exert anti-infection activity, lessen nerve damage, and promote nerve repair. However, they do not always provide protection, particularly when microglia-mediated neuroinflammation is challenging to control (Joers et al., 2017; Bai et al., 2020; Xia et al., 2020). In this condition, neurotoxic substances such as reactive oxygen species (ROS), nitric oxide (NO), interleukin-1β (IL-1β), and tumor necrosis factor-α (TNF-α) might be produced. Therefore, understanding the detailed mechanism of phenotypic transformation of microglia under physiological or pathological conditions is of great significance for diagnosing and treating many central nervous system diseases. Prior to this objective, identifying peripheral monocytes, intracranial boundary macrophages, and microglia is of utmost importance. Fortunately, this wish has gradually come true with the advancement of single-cell sequencing and microscopic imaging technology, and some microglia-specific molecular markers have been gradually revealed (Butovsky et al., 2014; Bennett et al., 2018). At the same time, the microglial phenotypic transformation becomes clear with this technology’s support during the disease’s occurrence, development, and outcome (Keren-Shaul et al., 2017).

Due to the development of neuroscience research, the research literature in the field of microglia is also increasing rapidly (Sierra et al., 2019). There is frequently no way to start with such a large body of literature for new researchers. Bibliometrics is an excellent way to solve this problem (Chen, 2006; Hou et al., 2022). Mathematical statistical methods in bibliometrics are often used to systematically explore and evaluate a field’s intellectual base, hotspots, and directions. By using the method of bibliometrics, we can make quantitative statistics and visual analyses of cooperative networks of disciplines, countries, institutions, authors, and journals in the research field (Agarwal et al., 2016). We can also carry out co-word, co-occurrence, co-citation, and coupling analysis of keywords, terms, and references and construct a visual knowledge graph (Chen et al., 2014, 2018). It is helpful for researchers to quickly grasp the basic situation of a specific research field and the hot spots, key points, and difficulties in a specific direction, which traditional reviews cannot do. Bibliometrics is gradually becoming known to the general public and used in literature analysis due to the rapid growth of scientific research literature data. We construct the intellectual foundation of microglia research in the 20 years from 2002 to 2021 using the two-core bibliometrics software CiteSpace and VOSviewer and aim to identify its development path, research trend, and challenges. First, we make a statistical analysis of disciplines, journals, authors, countries, institutions, co-cited authors, and co-cited journals to understand the basic information in this field; secondly, we carry out clustering and burstness analysis of keywords to explore the research direction, hotspots, and evolution over time in the field. Finally, through the co-citation analysis of the references, we understand the core literature in the field on the one hand and the knowledge structure and dynamic changes in the field on the other.

## Materials and methods

### Data search and download

The literature data for bibliometric analysis is retrieved from the Web of Science Core Collection. We set the following search restrictions: title “microglia” OR “microglial,” publication date “2002-01-01” to “2021-12-31,” document types “article” or “review article,” language “English.” We found 12,815 relevant documents on microglia using the above search method and 12,813 documents after CiteSpace removed duplicates.

### CiteSpace

CiteSpace is an information visualization software developed by Chen (2006), a famous bibliometrics expert, based on citation analysis theory. Numerous functions, including cooperative network analysis, co-word analysis, co-citation analysis, and others, can be realized by it (Chen, 2004; Li and Liu, 2020). At the same time, its unique betweenness centrality and burstness analysis have attracted much attention. Betweenness centrality is when an entity connects the content of different groups, indicating that the entity plays a turning point. “Burstness” denotes an abrupt rise in the frequency of a keyword or numerous citations over time in a body of literature; this definition also applies to the abrupt rise in the number of articles published by countries/regions, organizations, or authors (Chen et al., 2014). Betweenness centrality and burstness analysis are very helpful for us to quickly understand the evolution of knowledge structures, milestone papers, research hotspots and frontiers, and emerging topics. The time slice for our analysis is set to 1 year, the *g*-index to *k* = 25, and the citation analysis to *k* = 20. Each analysis selects a node type, and the clustering analysis uses the LLR algorithm. We use CiteSpace to perform subject category co-occurrence analysis, literature clustering, keyword clustering, and the burstness analysis of countries, institutions, keywords, and references.

### VOSviewer

Leiden University in the Netherlands developed this software for creating, visualizing, and exploring literature data (van Eck and Waltman, 2010). Its simple interface and unique overlay view are well received. We use it for visual analysis of country/regional cooperation networks, institutional cooperation networks, author cooperation networks, and keyword co-word network graphs (Ding and Yang, 2020; Chen et al., 2021; Liu and Avello, 2021). The parameters were as follows: “method” was set to “association strength” (country) or “LinLog/modularity” (institution, author, keyword); “attraction,” “repulsion,” and “resolution” were adjusted according to the content.

It should be noted that the averaged normalized number of citations (Avg. norm. Citations, i.e., citation intensity described in this manuscript) of a document equals the number of citations of the document divided by the average number of citations of all documents published in the same year and included in the data that is provided to VOSviewer. The normalization corrects the fact that older documents have had more time to receive citations than more recent documents.

Excel is used to export the data produced by CiteSpace and VOSviewer analysis for further sorting, filtering, and analysis. When conducting keyword analysis, we only choose the author’s keywords, which are sufficient even though they are not exhaustive. We standardized the extracted keywords by (1) combining words with the same meaning so that they are treated as synonyms in the software, (2) removing duplicates, and (3) deleting unintentional words (such as numbers).

## Results

### Publication trends, subject categories, and journals

**Figure 1:**
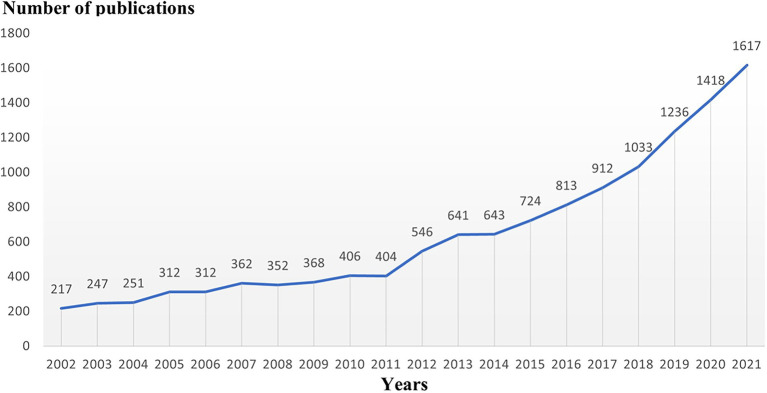
Trends of publications of microglial research between 2002 and 2021.

**Figure 2:**
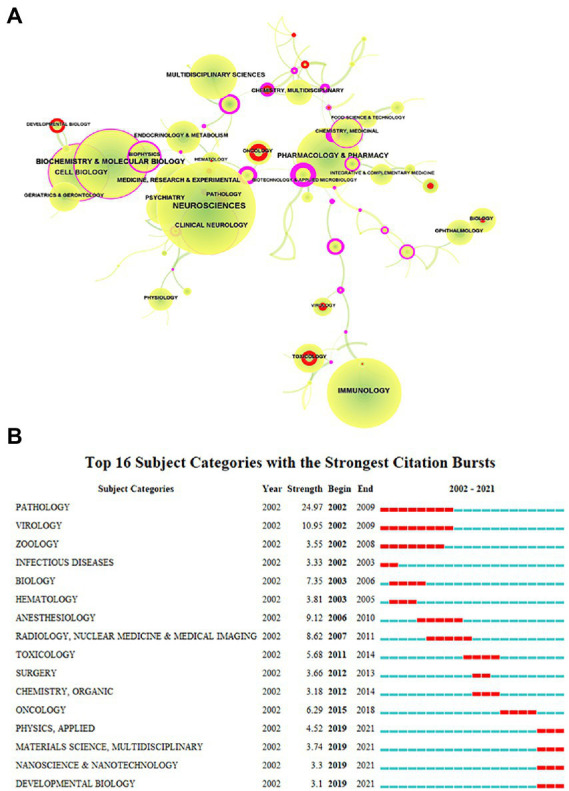
Subject categories of publications analyzed by CiteSpace. **(A)** The size of the node represents the number of publications, the link represents the degree of connection between disciplines, the color of the node and link represents different years, the color from green to yellow represents 2002 to 2021, red represents the intensity of burst, and purple represents betweenness centrality. **(B)** The burstness detection of the subject category by CiteSpace, “strength” represents the intensity of the burst, “begin” represents the beginning year of the burst, “end” indicates the ending year of the burst, red dotted line indicates the duration of the burst. The blue line indicates the entire period from 2002 to 2021.

When evaluating the advancement of scientific investigation, which partly reflects the growth of knowledge in this field, the annual volume of articles is a significant factor to consider. Figure 1’s depiction of the published literature’s time distribution revealed that the number of articles about microglia had continued to rise, particularly since 2011, when it spiked sharply and grew significantly through 2021. We next explored the discipline attributes in the field of microglia research. As seen in Figure 2A, research on microglia is primarily focused on five fields: neurosciences, immunology, biochemistry and molecular biology, pharmacology and pharmacy, and cell biology. However, a discipline burstness analysis (a total of 16 bursts are detected; Figure 2B) reveals that early studies concentrated on pathology and virology, then emerged in anesthesiology and radiology, nuclear medicine, and medical imaging, and finally appeared in toxicology and surgery. Recently, it has been chiefly seen in physics, material science, and developmental biology (Figure 2B).

We used VOSviewer to make statistics on journal information. Twelve Thousand Eight Hundred Thirteen articles are published in 1,336 journals. The top 10 journals are chosen based on the number of articles, as shown in Table 1. Glia and J Neuroinflamm account for about 1/10 of the articles, followed by J Neurochem, Brain Behav Immun, J Neurosci, J Neuroimmunol account for another 1/10. Among these journals, 5 in Q1, 3 in Q2, 2 in Q3, and 7 have an impact factor of more than 5. Then we analyze the cited journals. The top 10 co-cited journals have all been cited more than 10,000 times. As shown in Table 1, J Neurosci, Glia, P Natl Acad Sci USA, J Biol Chem, Nat Neurosci, J Immunol Science, J Neuroinflamm, Nature, and J Neurochem are the top 10 highly cited journals, of which there are 7 in Q1, 3 in Q2 and 2 with impact factors over 60.

**Table 1:** The top 10 journals and co-cited journals in microglia research field.

Journal	Count	IF (2021)	JCR	Co-cited Journal	Count	IF (2021)	JCR
Glia	626 (4.89%)	8.073	Q1	J Neurosci	31,790 (4.40%)	6.709	Q1
J Neuroinflamm	591 (4.61%)	9.587	Q1	Glia	24,488 (3.39%)	8.073	Q1
J Neurochem	268 (2.09%)	5.546	Q2	P Natl Acad Sci USA	16,716 (2.32%)	12.779	Q1
Brain Behav Immun	266 (2.08%)	19.227	Q1	J Biol Chem	14,893 (2.06%)	5.486	Q2
J Neurosci	262 (2.04%)	6.709	Q1	Nat Neurosci	14,762 (2.05%)	28.771	Q1
J Neuroimmunol	256 (2.00%)	5.426	Q2	J Immunol	14,386 (1.99%)	5.426	Q2
Plos One	235 (1.83%)	3.24	Q2	Science	14,051 (1.95%)	63.714	Q1
Front Cell Neurosci	224 (1.75%)	6.147	Q1	J Neuroinflamm	13,640 (1.89%)	9.587	Q1
Neurosci Lett	221 (1.72%)	3.197	Q3	Nature	13,543 (1.88%)	69.504	Q1
Neuroscience	199 (1.55%)	3.708	Q3	J Neurochem	13,254 (1.84%)	5.546	Q2

### Country and institutional distributions of microglia research

In this study, the cooperative network map of countries/regions in this field was made using VOSviewer software. The results show that 91 countries have participated in this field. The top 10 countries/regions in terms of the number of articles (Table 2) are the United States (3983), People R China (3395), Germany (1233), Japan (1175), South Korea (1175), England (682), Canada (629), Italy (5505), France (430), and Spain (420). Among them, the first four countries accounted for 67.38% of the total articles.

**Table 2:** The top 10 countries/regions involved in microglia research field.

Rank	Country	Publication	Centrality	Avg. norm. Citations	Total link strength
1	United States	3,983 (31.09%)	0.34	1.38	2,118
2	Peoples R China	3,395 (26.50%)	0.13	0.78	933
3	Germany	1,233 (9.62%)	0.34	1.40	1,168
4	Japan	1,175 (9.17%)	0.08	0.85	453
5	South Korea	929 (7.25%)	0.15	0.66	240
6	England	682 (5.32%)	0.1	1.64	842
7	Canada	629 (4.91%)	0.12	1.28	492
8	Italy	550 (4.29%)	0.05	1.11	436
9	France	430 (3.36%)	0.11	1.16	521
10	Spain	420 (3.28%)	0.07	1.06	376

**Figure 3:**
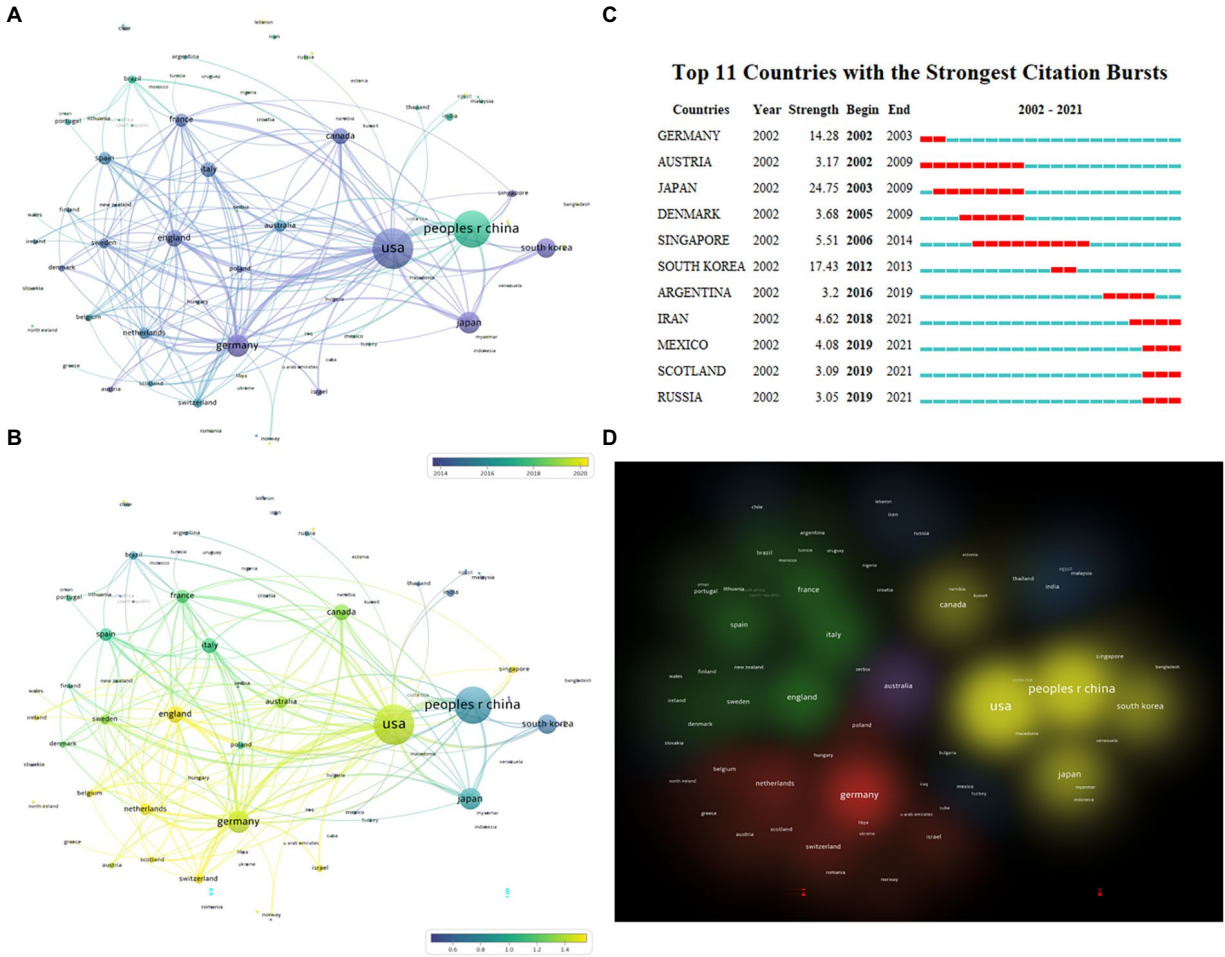
Distribution of publications from different countries/regions. **(A)** Overlay view of countries/regions cooperation network with average publication year, which is indicated by color from dark blue to bright yellow (VOSviewer). **(B)** Overlay view of countries/regions cooperation network with normalized average citations. Dark blue means lower citations, while bright yellow means higher citations (VOSviewer). **(C)** The burstness detection of national publications by CiteSpace, “strength” represents the intensity of the burst, “begin” represents the beginning year of the burst, “end” indicates the ending year of the burst, red dotted line indicates the duration of the burst. The blue line indicates the entire period from 2002 to 2021. **(D)** Cluster view of cooperative relationships between countries, with the same color representing closely cooperative countries (VOSviewer).

The number of articles published does not fully represent a country’s contribution to a specific field, so on this basis, we explore the average publication time and citation intensity (Figure 3) of documents in each country/region. As can be seen from the figure, the average publication time of most of the top countries is around 2014, while People R China is around 2017 (Figure 3A). According to the citation intensity of articles in various countries, it is found that the citation intensity is higher in the United States, Germany, England, and the Netherlands, slightly lower in Canada, Italy, and France, and poor in People R China, Japan, and South Korea (Figure 3B; Table 2). We continue to analyze the betweenness centrality (which can measure the country’s role in the knowledge dissemination process) of each country. Betweenness centrality greater than 0.1 is considered to play a more critical role, while the United States, Germany, South Korea, People R China, Canada, France, and England are all greater than 0.1 (Table 2). CiteSpace was used to carry out burstness detection. We discovered that 11 countries/regions had reached the stage of a surge in the number of bursts (Figure 3C). We also discovered that the volume of articles from Iran, Mexico, Scotland, and Russia had significantly increased over the past 2 years, indicating that these countries/regions may go on to play a more prominent role in this field (Figure 3C). The clustering view shows much cooperation between countries, with the United States-People R China-Japan-South Korea, Germany-Netherlands-Switzerland, and England-France-Spain-Italy constituting the three largest cooperation groups (Figure 3D).

**Figure 4:**
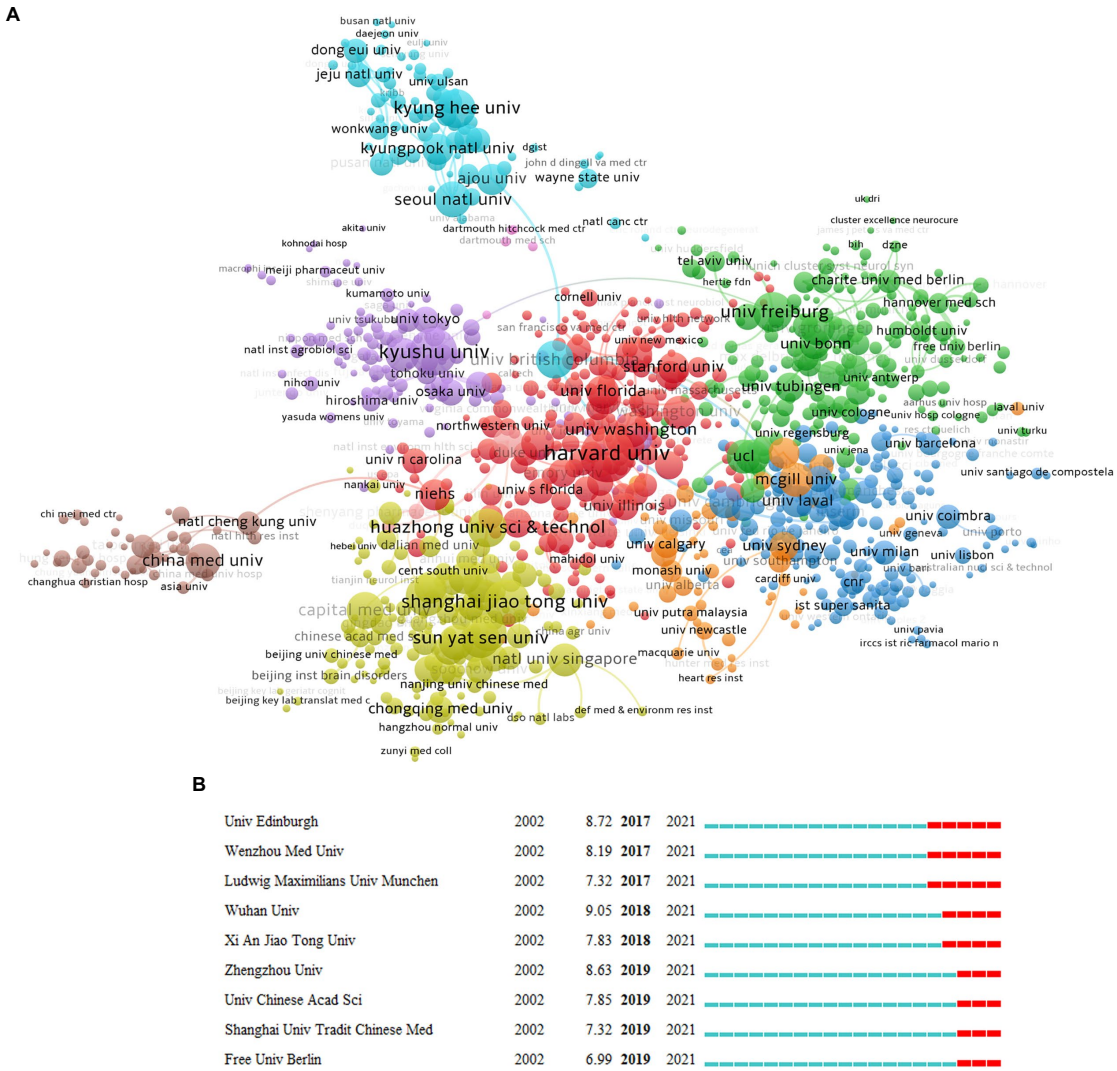
Distribution of publications from different institutions. **(A)** The network diagram of institutional cooperation, in which the same color represents a closely cooperative organization (VOSviewer). **(B)** The burstness detection of the number of institutional publications (lasting until 2021) by CiteSpace, “strength” represents the intensity of the burst, “begin” represents the beginning year of the burst, and “end” indicates the ending year of the burst, red dotted line indicates the duration of the burst. The blue line indicates the entire period from 2002 to 2021.

Then we use a similar method to analyze the research institutions; the top 10 institutions are listed in Supplementary Table S1, which are Harvard Univ (267), Kyushu Univ (204), Shanghai Jiao Tong Univ (193), Fudan Univ (153), Univ Freiburg (145), Chinese Acad Sci (140), Kyung Hee Univ (138), Sun Yat Sen Univ (137), Huazhong Univ Sci and Technol (136), and Univ British Columbia (134). Regarding betweenness centrality, Harvard Univ (0.15) ranks first, followed by Kyushu Univ (0.07). In terms of citation intensity, Harvard Univ (2.67) and Univ Freiburg (2.29) are cited most intensively (Supplementary Table S1). According to the cooperative network map (Figure 4A), there are eight large cooperative groups, with Harvard Univ, Univ Freiburg, Shanghai Jiao Tong Univ, Kyushu Univ, Kyung Hee Univ, China Med Univ, Cambridge Univ, and McGill Univ. as the core. We also used CiteSpace for burstness detection, which developed rapidly after 2017. We found that 9 institutions met the criteria (Figure 4B), with the top three being Wuhan Univ, Zhengzhou Univ., and Univ. Edinburgh, respectively, suggesting that these institutions have more potential for development.

### Author contributions in the field of microglial research

**Figure 5:**
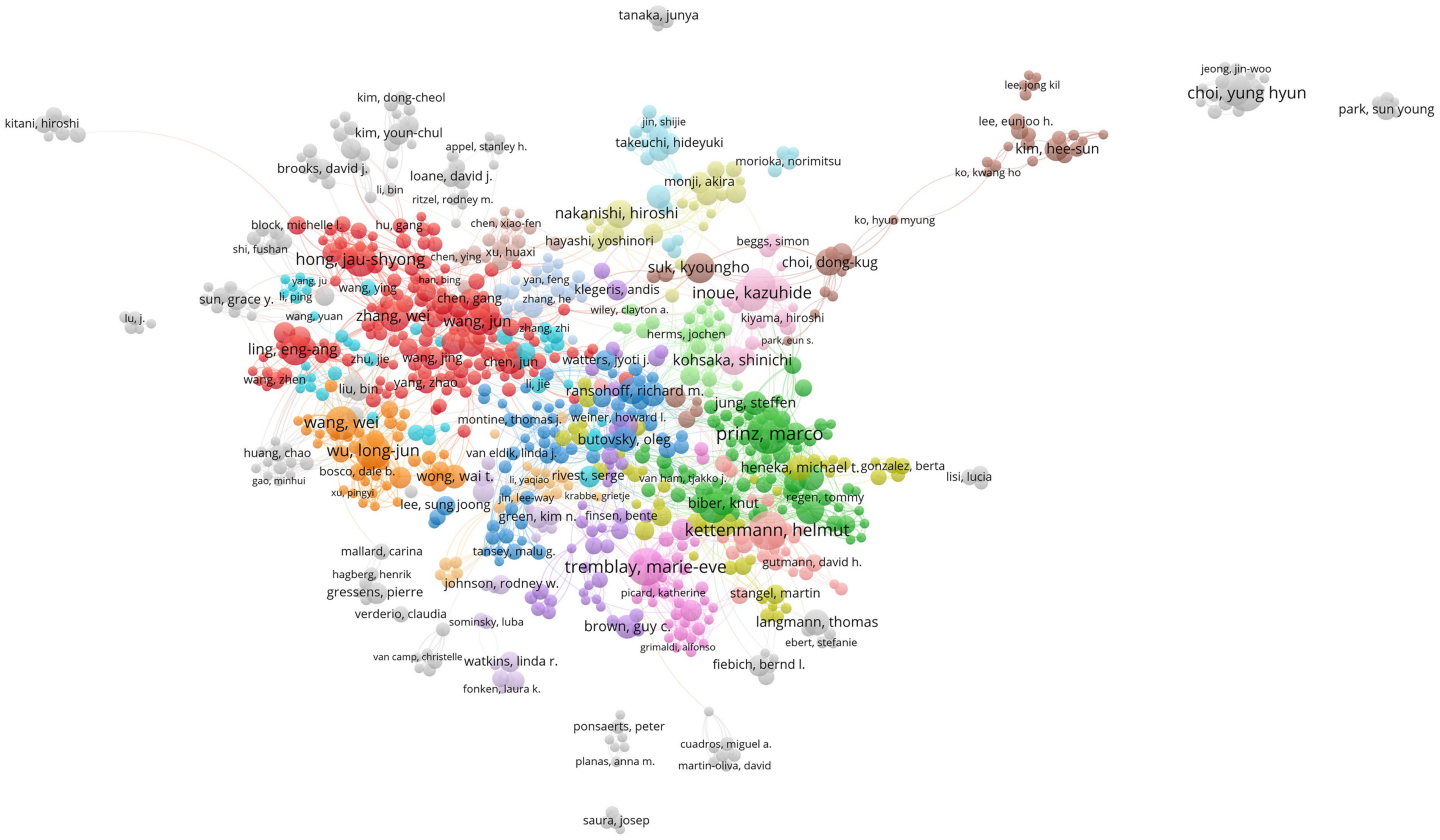
The co-occurrence map of authors in the microglial research field (VOSviewer). The node’s size reflects the co-occurrence frequency of the authors, the link reflects the co-occurrence relationship between the authors, and different colors represent the clustering results of the cooperative network, and the closer the relationship is with the same color.

We use the statistical data from VOSviewer as the foundation because Web of Science and CiteSpace base their counts on name abbreviations, which are significantly impacted by duplicate names. The top 10 authors are listed on the left side of the table (Table 3). They are Prinz Marco (70), Kettenmann Helmut (62), Tremblay Marie-Eve (59), followed by Choi Yung Hyun (53), Wu Long-Jun (53), Inoue Kazuhide (51), Wang Wei (47), Hong Jau-Shyong (46), Tsuda Makoto (46), Kim Gi-Young (45). Among the 10 authors, Prinz Marco (3.85) and Kettenmann Helmut (2.40) also have a higher citation intensity indicated by Avg. norm. citations. We also list the top 10 authors in citation intensity (Table 3): Bennett Mariko L. (14.70), Keren-Shaul Hadas (13.81), Barres Ben A. (12.75), followed by Liddelow Shane A. (12.63), Panicker Nikhil (11.93), and Bohlen Christopher J. (11.75). These authors may not have many papers, but their research may have an important impact on the field. We have also analyzed the authors in the references, called the “cited authors”, namely Streit Wj (2797), Hanisch Uk (1869), Ransohoff Rm (1813), Perry Vh (1730), Nimmerjahn A (1722), and Mcgeer Pl (1642). To explore the cooperation between the authors, we have constructed a cooperative network graph of the authors. As shown in Figure 5, different colors represent different clusters. We can see that Prinz Marco (green), Kettenmann Helmut (light red), Tremblay Marie-Eve (pink), Inoue Kazuhide (light pink), Ransohoff Rm-Butovsky O (blue), Hong Jau-shyong-Zhang Wei (red), Wu Longjun (orange), Nakanishi Hiroshi (yellow green), and Kim Hee-sun (brown) has formed a large research group.

**Table 3:** The top 10 authors and co-cited authors involved in microglia research field.

Author	Documents	Avg. norm. Citations	Author	Avg. norm. Citations	Cited Author	Citations
Prinz Marco	70	3.85	Bennett Mariko L.	14.70	Streit Wj	2,797
Kettenmann Helmut	62	2.40	Keren-Shaul Hadas	13.81	Hanisch Uk	1869
Tremblay Marie-Eve	59	1.81	Barres Ben A.	12.75	Ransohoff Rm	1813
Choi Yung Hyun	53	1.72	Liddelow Shane A.	12.63	Perry Vh	1730
Wu Long-Jun	53	0.58	Panicker Nikhil	11.93	Nimmerjahn A	1722
Inoue Kazuhide	51	1.23	Bohlen Christopher J.	11.75	Mcgeer Pl	1,642
Wang Wei	47	0.69	Amit Ido	9.67	Block Ml	1,641
Hong Jau-Shyong	46	1.54	David Eyal	8.98	Kettenmann H	1,555
Tsuda Makoto	46	1.21	Stevens Beth	7.88	Butovsky O	1,523
Kim Gi-Young	45	0.54	Franklin Robin J. M.	7.80	Ginhoux F	1,441

### Knowledge domains, evolutions, and emerging trends

Keywords are the generalization of a field of research. To explore the intellectual structure and dynamic changes in microglia research in the past 20 years, we used VOSviewer and CiteSpace for co-word analysis and clustering. The keywords in the top 20 are shown in Table 4 (keywords A, here, we have removed “microglia”); we can see that the primary research has focused on neuroinflammation. The second is to study the phenotypic changes, cytokine release, phagocytic activity, and cytotoxicity of microglia in different neurodegenerative diseases (AD, PD, etc.).

**Table 4:** The top 20 keywords involved in microglia research field.

Keywords (A)	Occurrences	Cluster	Keywords (B)	Occurrences	Avg. norm. Citations	Cluster
Neuroinflammation	3,301	1	Chronic stress	25	1.87	2
Alzheimer’s disease	899	6	Priming	28	1.81	2
Lipopolysaccharide	656	3	m2	32	1.72	1
Microglial activation	543	1	exosomes	59	1.70	1
nf-kb	542	3	pk11195	28	1.64	6
Astrocyte	516	4	Synaptic pruning	33	1.63	2
Neurodegeneration	483	1	Glycolysis	26	1.54	8
Phagocytosis	456	6	m1/m2 phenotype	162	1.51	1
Cytokines	446	5	Human	34	1.48	5
Macrophages	428	5	Exosome	27	1.46	1
Nitric oxide	381	3	Extracellular vesicles	51	1.45	1
Neuroprotection	355	1	Senescence	32	1.43	2
Beta-amyloid	347	6	Ageing	29	1.42	1
Parkinson’s disease	342	1	csf1r	27	1.41	5
tnf alpha	303	3	Neuropathology	34	1.38	6
Apoptosis	266	8	Pet imaging	106	1.37	6
mapk	256	3	Sex differences	39	1.35	2
Microglia polarization	250	1	Heterogeneity	27	1.34	5
Oxidative damage	238	1	Regeneration	31	1.32	7
Hippocampus	228	2	Neurodegeneration	483	1.31	1

**Figure 6:**
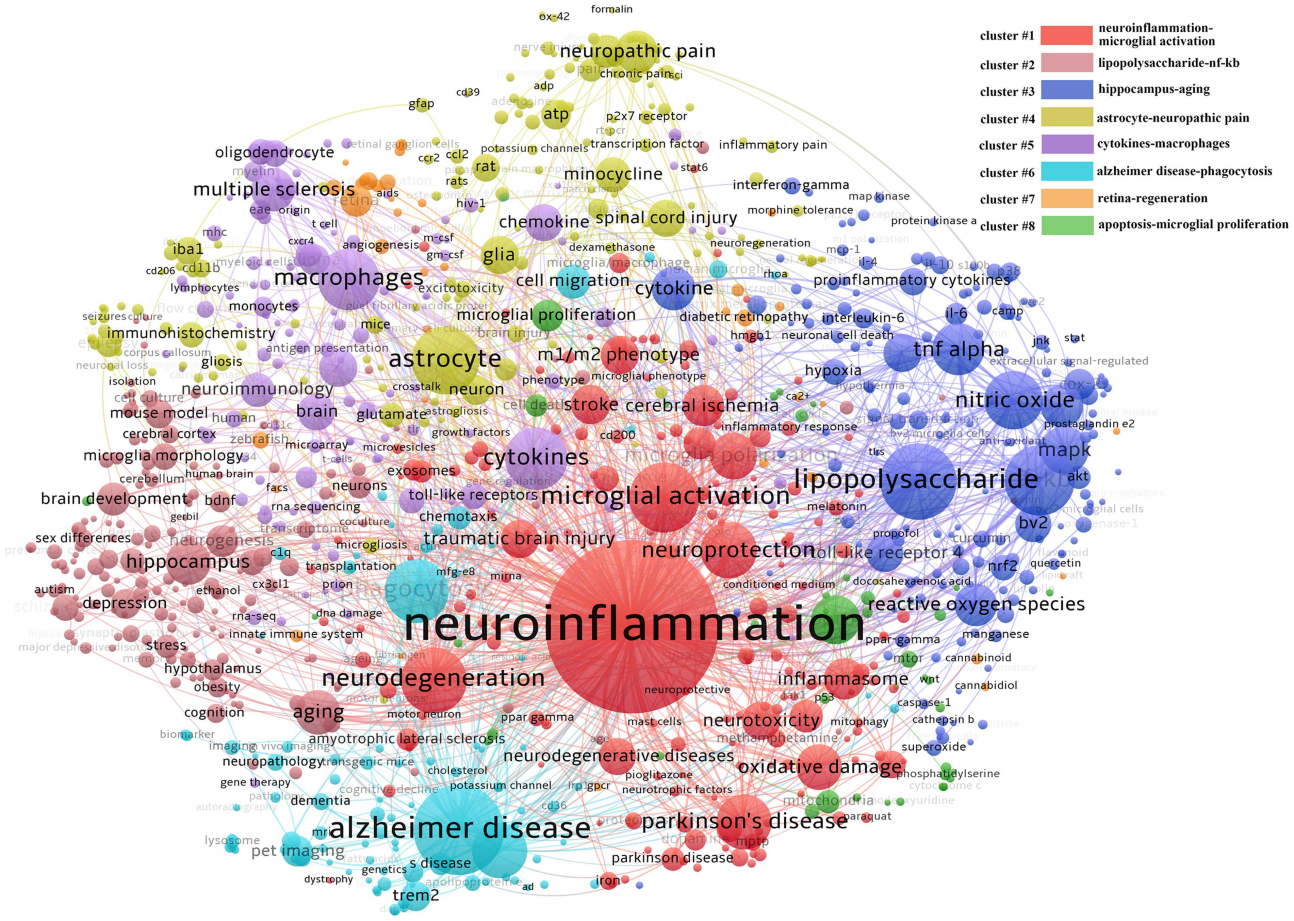
Keywords co-word network and clustering in the field of microglia research. The node size reflects the co-word frequency, the link represents the co-word intensity, different colors represent the clustering results of the co-word network, and the closer the relationship is with the same color.

According to the results of VOSviewer clustering we got 8 clusters (Figure 6) labeled with the top two keywords in each cluster. The first group is neuroinflammation and microglial activation (cluster#1). As the largest group it involves a broad spectrum of diseases such as neurodegenerative diseases TBI and stroke. The main research content involved is the M1/M2 phenotype, microglial polarization and the associated neuroprotection or neurotoxicity functions. The second group is lipopolysaccharide and nf-κb signal pathway (cluster#2); this group was like cluster#1. Other keywords in the group such as NO, TNF-α toll-like receptor, mitogen activated protein kinase (MAPK), and ROS suggest that this group mainly studies the activators and signaling pathways involved in the inflammatory activation of microglia as well as the resulting inflammatory and oxidative stress damage. The third group is hippocampus and aging (cluster#3) which also involves the keywords brain development, nerve regeneration, obesity, depression, gender difference, and hypothalamus. Unlike the first two groups this group explores some functional diseases mainly the functional heterogeneity of microglia and its effects on synaptic pruning and nerve regeneration. The fourth group is astrocytes and neuropathic pain (cluster#4) suggesting that the interaction between microglia and astrocytes greatly influences pain. At the same time combined with other keywords in this group such as minocycline, spinal cord injury, and neurons it can be speculated that this group is involved in the mechanism and treatment of neuropathic pain caused by diseases such as spinal cord injury and implies the potential for using minocycline

The fifth group is cytokines and macrophages (cluster#5), which also involves keywords such as multiple sclerosis, oligodendrocytes, neuro-immunity, and chemokines. It suggests macrophage-oligodendrocyte interaction plays a vital role in the pathogenesis and progression of multiple sclerosis. The sixth group is AD and phagocytosis (cluster#6), and the keywords amyloid protein, Trem2, and pet imaging are also involved in the group, indicating that AD has received extensive attention. Currently, the disease is related to the deposition of amyloid protein, so the study of the phagocytosis mechanism of microglia is the mainstream direction. At the same time, the detection and observation of amyloid protein are important for disease diagnosis and therapeutic effect evaluation, so detecting amyloid protein by pet imaging under various molecular markers is very important. The seventh group is the retina and regeneration (cluster#7), with relatively little but professional research. The research object is the retina; combined with zebrafish, diabetic retinopathy, age-related macular degeneration, and muller cells, we can see that the primary research content of this group is the role of retinal microglia in various retinal-related diseases and the exploration of improving nerve regeneration by regulating microglia. The eighth group is the most miniature set, which is related to apoptosis and proliferation (cluster#8), and its group also involves mitochondria, glycolysis, OGD, cell cycle, and cell survival, suggesting that this group is mainly involved in the internal molecular regulation of microglial survival, proliferation, or energy metabolism.

**Figure 7:**
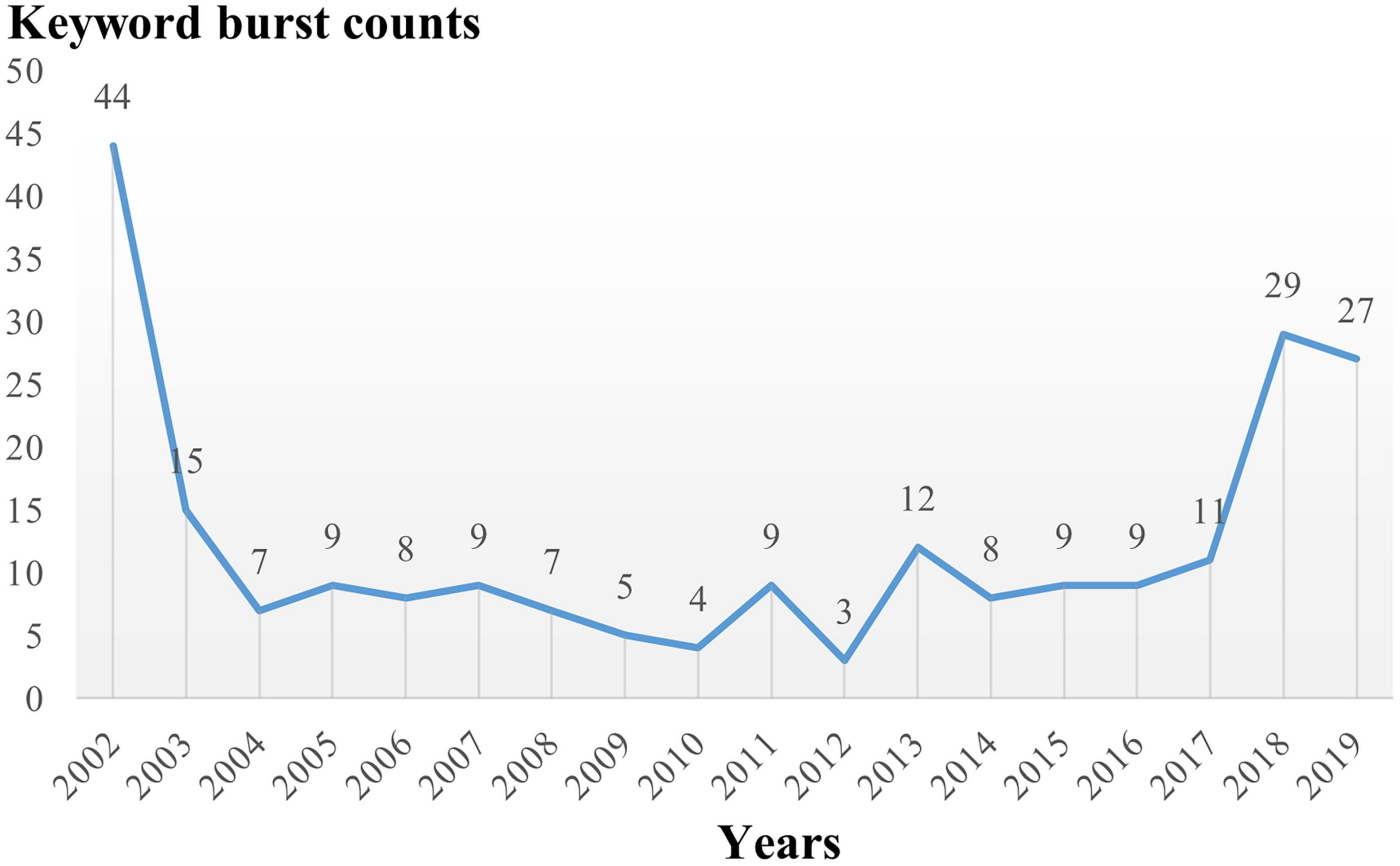
The number of burst keywords distribution over time.

**Figure 8:**
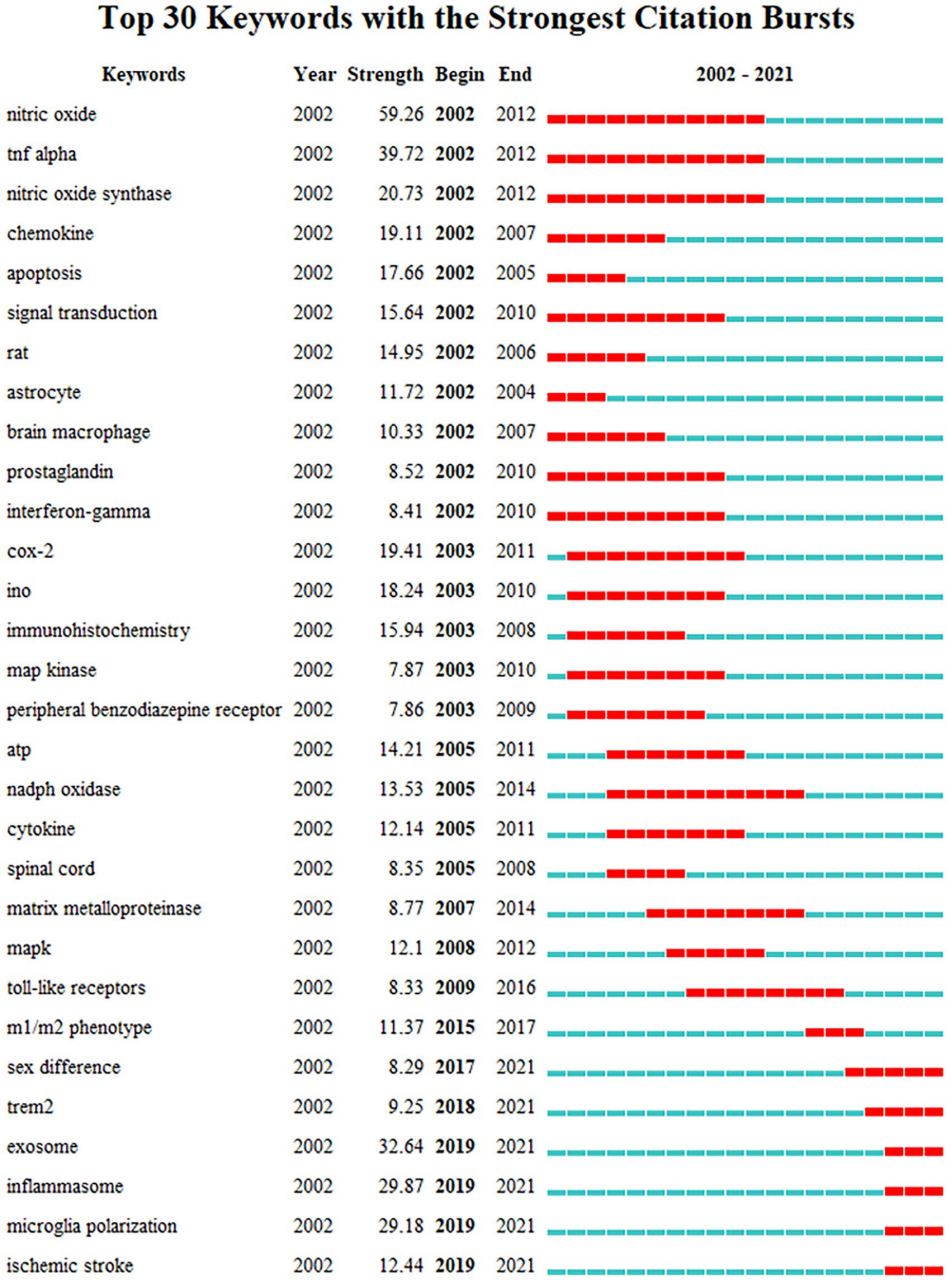
The top 30 burst of keywords analyzed by CiteSpace, “strength” represents the intensity of the burst, “begin” represents the beginning year of the burst, “end” indicates the ending year of the burst, red dotted line indicates the duration of the burst. The blue line indicates the entire period from 2002 to 2021.

The clustering result of CiteSpace is 9 clusters in timeline view (Supplementary Figure S1), similar to that of VOSviewer but different in that apoptosis and glycolysis clustering are classified into the same group while exosomes belong to the first group in VOSviewer. This difference suggests that the clustering results for these keywords have a high degree of credibility. The timeline shows that each cluster was studied in 2002 and continued to 2021 after continuous development. To further explore the keywords’ evolution, burstness detection was performed. We finally got 228 bursts and made a chart based on the number of burst words each year. According to the graph (Figure 7), there was a spike in the number of keywords from 2002 to 2003, followed by a long period of silence from 2004 to 2017, and then a sharp increase after 2017. The top 30 keywords were then intercepted (Figure 8). We discovered that the 2002 keywords that suddenly increased were primarily related to the inflammatory process; the 2003–2009 keywords are the expansion of the 2002 keywords. Since 2015, there has been a significant shift in focus away from the typical study of microglia pro-inflammatory activation toward the regulation of the M1/M2 phenotype and a greater focus on the gender differences of microglia, phagocytosis (Trem2), and the exosome. We also explored the keywords that lasted until 2021 and found that retinal generation/phenotype remained hot for 5 years, while sex difference/Trem2/zebrafish/RNA-seq/exosome/inflammasome/microglial polarization was the focus of research in the past 5 years (Supplementary Figure S2).

We further investigate which keywords have a high citation intensity, which may indicate how significant the literature containing the keyword is. It also suggests that this article’s author is an authority on the subject. These keywords, which included exosome, microglial polarization, pet imaging, CSF1R, synaptic pruning, glycolysis, microglial heterogeneity, and gender differences, were discovered to be primarily concentrated in cluster 1/2/5/6, with a focus on human, chronic stress, aging, neurodegenerative diseases, and nerve regeneration (Table 4, keywords B).

### Co-cited analysis of references

**Figure 9:**
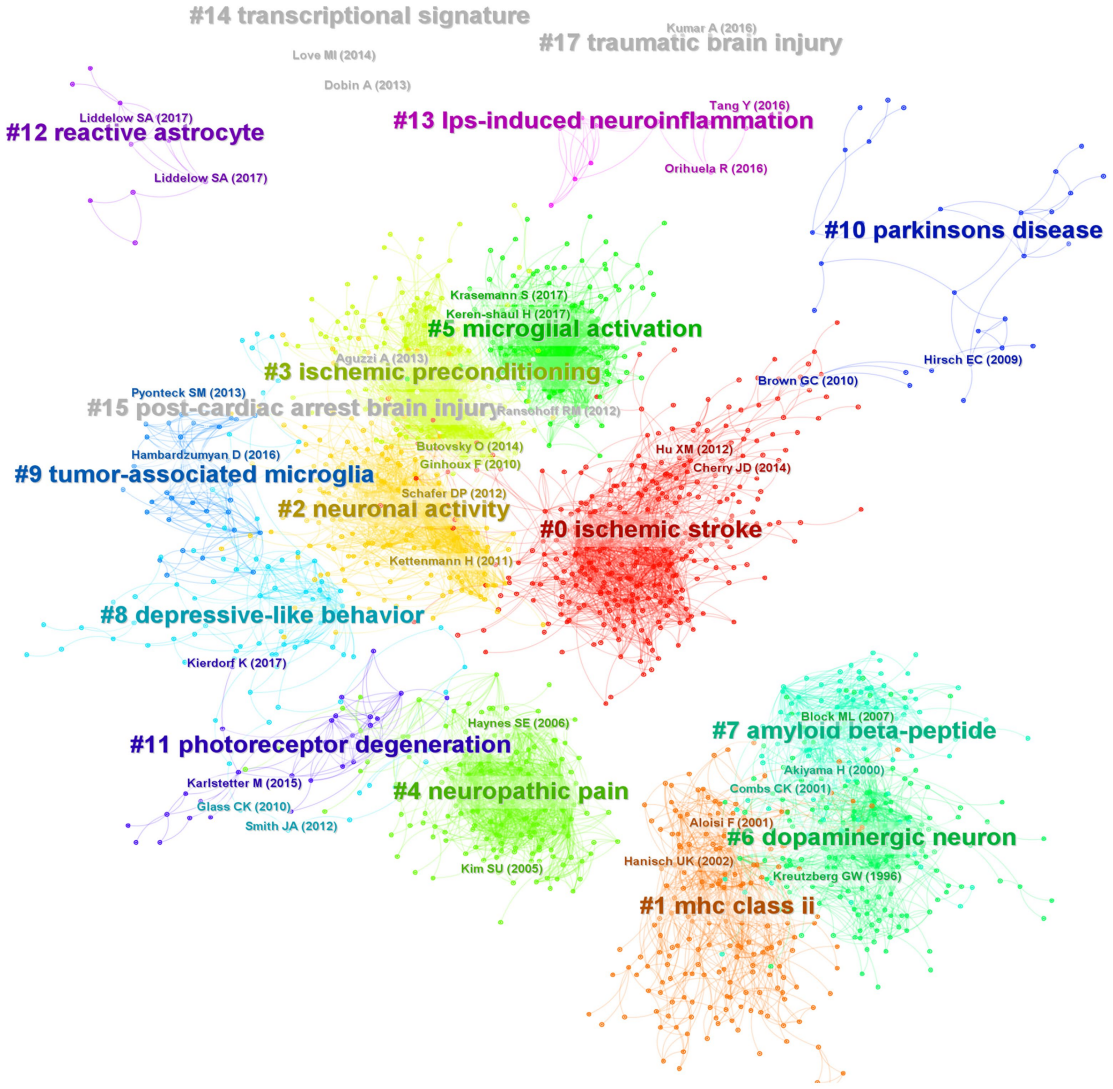
Co-citation network and clustering constructed by references. Each node represents a reference, the link between nodes represents the co-citation intensity, and different colors represent different clusters.

Highly cited literature is an essential source of knowledge in a research field, which reflects the research frontier and level in this field and is an important basis for exploring the research context and development direction. According to the analysis of cited literature by CiteSpace, there are 284,695 articles, 1966 nodes, and 12,527 edges. After clustering the cited networks, 17 clusters were obtained (Figure 9; Supplementary Table S2) and labeled with title words or keywords from the citing articles. Silhouette >0.7 in these clusters denotes the plausibility of the clustering findings. From the table, all the clusters are good. These clusters are mainly disease-related (stroke, neuropathic pain, AD, PD, depression, glioma, retina, cardiac arrest, TBI), cell communication-related (synapse, astrocytes), research technology-related (microglial depletion), and mechanism-related (glycolysis, DNA methylation). We further analyzed this cluster’s timeline map (Supplementary Figure S3) and found that stroke, chemokine, neuropathic pain, PD, and AD started earlier. Then came the synaptic pruning of microglia, followed by microglial depletion, depression, glioma, and retina.

**Figure 10:**
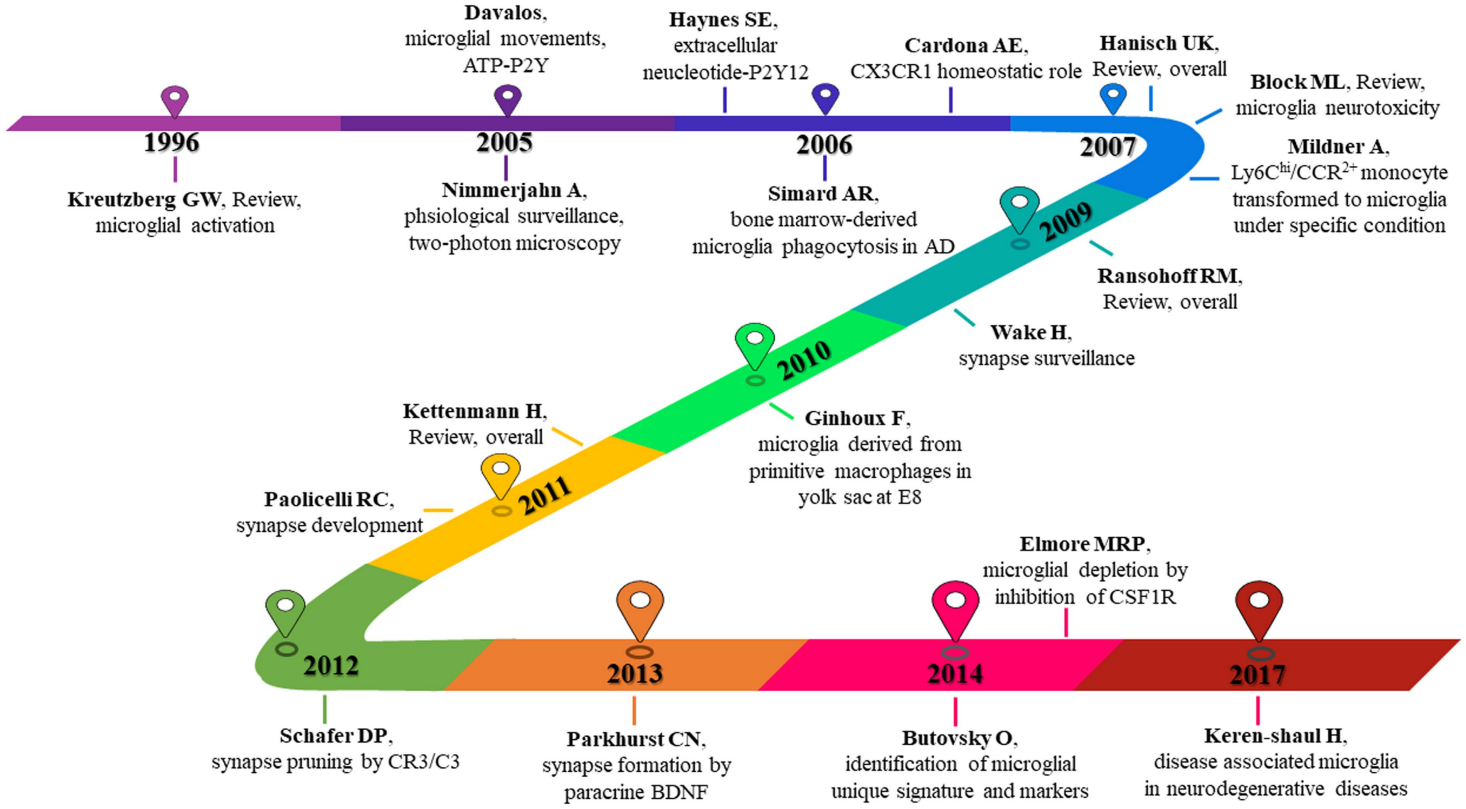
19 high-impact articles exhibited in the time chart. Nineteen articles were judged comprehensively by the number of publications, burstness, betweenness centrality, and sigma value. The figure lists the author, year, and main findings.

In order to understand which articles have a significant influence on this field, we measure them on three dimensions: citation frequency, burstness, and sigma value. Sigma value is the comprehensive evaluation result of betweenness centrality and burstness. Therefore, we use the sigma value to include betweenness centrality attributes. After selecting and summarizing the top 10 articles for each dimension, 19 influential articles were obtained and combined into one table or graph (Table 5; Figure 10). Among them, two articles by Ginhoux F 2010 in Cluster#3 and Keren-Shaul H 2017 in Cluster#5 appeared in three dimensions, indicating their pivotal positions. The long-running controversy was finally resolved in 2010 by Ginhoux F, who, using the lineage tracing method, established that microglia descended from primitive macrophages of the yolk sac in E8. Keren-Shaul H 2017 indicated that microglia in AD changed from resting microglia to the intermediate and disease-related states through two sequential activations (Trem2-independent and Trem2-dependent) by single-cell sequencing, which may play a protective role in AD.

**Table 5:** The summarizations of 19 influential articles involved in microglia research field.

References	DOI	Cluster	Filter
Keren-shaul H, 2017, CELL, 169, 1,276	10.1016/j.cell.2017.05.018	5	C.C., Bur., Sig.
Ginhoux F, 2010, SCIENCE, 330, 841	10.1126/science.1194637	3	C.C., Bur., Sig.
Kettenmann H, 2011, PHYSIOL REV, 91, 461	10.1152/physrev.00011.2010	2	C.C., Bur.
Hanisch UK, 2007, NAT NEUROSCI, 10, 1,387	10.1038/nn1997	2	C.C., Bur.
Block ML, 2007, NAT REV NEUROSCI, 8, 57	10.1038/nrn2038	6	C.C., Bur.
Paolicelli RC, 2011, SCIENCE, 333, 1,456	10.1126/science.1202529	2	C.C., Sig.
Nimmerjahn A, 2005, SCIENCE, 308, 1,314	10.1126/science.1110647	2	Bur., Sig.
Davalos D, 2005, NAT NEUROSCI, 8, 752	10.1038/nn1472	2	Bur., Sig.
Wake H, 2009, J NEUROSCI, 29, 3,974	10.1523/JNEUROSCI.4363-08.2009	2	Bur., Sig.
Butovsky O, 2014, NAT NEUROSCI, 17, 131	10.1038/nn.3599	3	C.C.
Schafer DP, 2012, NEURON, 74, 691	10.1016/j.neuron.2012.03.026	2	C.C.
Parkhurst CN, 2013, CELL, 155, 1,596	10.1016/j.cell.2013.11.030	2	C.C.
Elmore MRP, 2014, NEURON, 82, 380	10.1016/j.neuron.2014.02.040	3	C.C.
Ransohoff RM, 2009, ANNU REV IMMUNOL, 27, 119	10.1146/annurev.immunol.021908.132528	2	Bur.
Kreutzberg GW, 1996, TRENDS NEUROSCI, 19, 312	10.1016/0166-2,236(96)10049-7	6	Bur.
Haynes SE, 2006, NAT NEUROSCI, 9, 1,512	10.1038/nn1805	4	Sig.
Mildner A, 2007, NAT NEUROSCI, 10, 1,544	10.1038/nn2015	0	Sig.
Cardona AE, 2006, NAT NEUROSCI, 9, 917	10.1038/nn1715	0	Sig.
Simard AR, 2006, NEURON, 49, 489	10.1016/j.neuron.2006.01.022	0	Sig.

C.C., denotes citation counts; Bur., denotes burstness intensity; Sig., denotes sigma value.

Hanisch UK 2007 in Cluster # 2 and Kettenmann H 2011 in Cluster # 2 provide comprehensive information about microglia’s origin, development, perception, and function. Block ML 2007 in Cluster # 6 reviews the literature on neuronal damage or death caused by the overactivation of microglia before 2007. Paolicelli RC 2011 in Cluster # 2 describes the involvement of microglia in synaptic pruning during nerve development. Using the two-photon imaging technique, Nimmerjahn A 2005 in Cluster # 2 observed microglia dynamically monitoring the surrounding environment. Additionally, microglia can move to tissue after tissue injury, including its processes and cell bodies, and this movement is mediated by ATP-P2Y, as reported by Davalos D. (2005) in Cluster # 2. Wake H 2009 in Cluster # 2 described how microglia could dynamically monitor synapses under physiological circumstances and clear damaged synapses during cerebral ischemia.

By comparing peripheral monocytes and microglia, Butovsky O. (2014) in Cluster # 3 discovered the distinct molecular phenotype of microglia and that TGF-β plays a crucial role in their development and is essential for *in vitro* culture. Schafer DP 2012 in Cluster # 2 confirmed that microglia could prune the synapses of neurons during development through the CR3/C3 signaling pathway, which depends on neuronal activity. Parkhurst CN 2013 in Cluster # 2 confirmed that microglia could promote learning-related synaptic formation by paracrine BDNF. Elmore MRP 2014 in Cluster # 3 described the importance of CSF1R in microglia survival. It proposed that PLX3397 can be used as an effective way to remove microglia, creating a convenient way for the study of microglia. Ransohoff RM 2009 in Cluster # 2 gave a detailed and complete review of microglia. Kreutzberg GW 1996 in Cluster # 6 reviewed microglial activators, signal pathways and cellular communication. Microglia P2Y12 receptor can sense extracellular nucleotide signals and then undergo morphological changes and migration, which may be the first step in microglial activation, according to Haynes SE 2006 in Cluster # 4. Mildner A 2007 in Cluster # 0 showed that only under specific host conditions can blood-derived Ly-6C^hi^CCR2^+^ mononuclear cells enter the brain and transform into microglia. The significance of microglia CX3CR1 for microglia homeostasis was discussed by Cardona AE 2006 in Cluster # 0. The lack of CX3CR1 will make microglia produce obvious neurotoxicity in both physiological and pathological conditions. Microglia derived from bone marrow are crucial for clearing plaque in AD, according to Simard AR 2006 in Cluster # 0.

## Discussion

In this paper, we systematically explore the articles related to the study of microglia from 2002 to 2021 by bibliometrics. The number of articles published grew slowly between 2002 and 2011. During these 10 years, 187 articles increased, a growth rate of 86.2%, while it multiplied after 2011, with an increase of 1,213 articles, a growth rate of 300%, and continued its momentum until 2021. People R China has contributed the most, barring the notable rise in the number of articles published by each nation after 2011 (Supplementary Figure S4), when we examine the proportion of articles from different countries/regions each year. In discipline distribution, neurology, immunity, and cell biology-related disciplines account for the most significant proportion. However, in recent years, interdisciplinary phenomena such as physics and material science cannot be ignored, which is expected to open a new direction for studying microglia. Twelve Thousand Eight Hundred thirteen articles published in 1336 journals, and the four journals of Glia, J Neuroinflamm, J Neurochem, J Neurosci ranked among the top 10 in terms of publication volume and total citation frequency analysis, suggesting that these four journals play an important role in microglial research. At the same time, well-known journals such as P Natl Acad Sci United States, Nat Neurosci, Science, Nature have also played an essential role.

The number of articles, citation intensity, burstness, betweenness centrality, and cooperative network can be used to measure the status and contribution of countries, institutions, and authors in a specific research field (Agarwal et al., 2016). According to the analysis of the countries, the United States, Germany, and England hold significant positions in all three major cooperative groups and are the leaders of these groups, suggesting that these three countries/regions are the main foundations for microglial research. Although People R China, Japan, and South Korea produce many papers, the number of citations is low, so they still need to continually raise the caliber and level of their research—which has enormous growth potential. From the analysis of institutions, Harvard Univ, Kyushu Univ and Shanghai Jiao tong Univ have the highest volume of papers. In comparison, only Harvard Univ has a betweenness center greater than 0.1, and Harvard Univ and Univ Freiburg are among the top two in terms of citation intensity. These dimensions suggest Harvard Univ is the core institution leading global microglial research. At the same time, the Univ Freiburg, Kyushu Univ, and Shanghai Jiao tong Univ are also in a critical position, and these institutions are also key nodes in the cooperative network. Regarding the authors, Prinz Marco and Kettenmann Helmut are both from Germany and hold prominent positions in terms of the volume of articles they have published and the frequency of citations, indicating that German researchers are influential in the field of microglia research. In the citation analysis, Streit Wj and Hanisch United Kingdom rank in the top two, which deserves the attention of researchers. It is also worth cautioning against the misunderstanding of the “Citation Burst” (like Figure 3C), which denotes an abrupt rise in the frequency of citations over time in a body of literature. Since Germany is always cited frequently and broadly, it is hard to get a citation burst according to the burstness algorithm. In this dimension, burstness is suitable for detecting the emerging group with a sudden increase in citation.

We tried to use CiteSpace and VOSviewer to construct the knowledge domains of microglia. First, we got 14,742 keywords and counted the changes in the number burst keywords over time. Except for the initial period of 2002–2003, when the volume of publications was lower, the burst of keywords was also lower. In contrast, as the number of articles rose sharply in 2012, the burst of keywords showed a transient increase before declining. Until the number of articles reached a steady growth stage after 2017, the number of burst keywords had increased significantly, suggesting that the burst of keywords has a certain lag. Then, from the perspective of specific content, neuroinflammation is not only the most extensive topic of microglial research but also a hot topic (Cherry et al., 2014; Leng and Edison, 2021). Numerous studies have been conducted on a variety of illnesses, including stroke (Bai et al., 2020), neurodegenerative diseases (Krasemann et al., 2017; Kwon and Koh, 2020; Leng and Edison, 2021), and TBI (Jassam et al., 2017), and they have all largely focused on the neuronal damage caused by inflammatory factors, oxidative stress, and lipopolysaccharide as a traditional activator model (Troutman et al., 2021). The concept of microglia polarization, or M1 and M2, has also gradually developed and has become the focus of microglial research (Ajmone-Cat et al., 2013; Byles et al., 2013; Joers et al., 2017; Bai et al., 2020; Mesquida-Veny et al., 2021). However, an increasing number of studies have revealed that the two phenotypes obtained from *in vitro* experiments are not distinct *in vivo* and are immutable in different diseases and periods of the same disease (Hu et al., 2015; Ransohoff, 2016; Mesquida-Veny et al., 2021). Microglia are frequently mixed, exhibiting both phenotypes. The idea of physiological state heterogeneity (Li et al., 2019; Cadiz et al., 2022), and gender differences (Fiore et al., 2022; Tansley et al., 2022; Ugidos et al., 2022) has slowly emerged in recent years, creating a new foundation for our complete understanding of microglia. At the same time, single-cell sequencing technology has also opened a new door for this (Butovsky et al., 2014). Disease-related microglia are gradually revealed (Keren-Shaul et al., 2017), which provides new ideas for solving this phenotypic problem. According to the clustering findings, the third group of research topics, which also includes the issue of nerve regeneration (Diaz-Aparicio et al., 2016), focuses on the origin, development, renewal, and death of microglia as well as their relationship with individual development and death (Ginhoux et al., 2010; Elmore et al., 2014; Oosterhof et al., 2018). The primary diseases involved in this problem are brain development (Bohlen et al., 2019), depression (Yirmiya et al., 2015), stress (Frank et al., 2018), autism (Sarn, 2021), and cognitive impairment (Feng et al., 2017). In this process, the effects of microglia on neurite development, synaptic remodeling, and nerve regeneration are the main research topics rather than neuroinflammation. Other than the two major research areas mentioned above, several diseases are being studied, including neuropathic pain (microglial-astrocyte communication) (Yu et al., 2019), multiple sclerosis (macrophage-oligodendrocyte communication) (Chiu et al., 2013), AD (Trem2-phagocytosis) (Boche and Gordon, 2022), and retinopathy (neurogenesis) (Okunuki et al., 2018; Wang et al., 2020), in which the unique role of microglia should be noted. Neuroinflammation still runs through the study of these diseases. Finally, it is worth mentioning that the minor groups, mitochondria, and glycolysis groups, suggest that attention has been paid to the transformation of energy metabolism in the activation process of microglia, which is called metabolic reprogramming (Baik et al., 2019; Bernier et al., 2020; Gu et al., 2021; Luo et al., 2021). It might also be a reference point for microglia with abnormal phenotypic change and function. According to research hotspots in recent years, the extraction of keywords, and protruding words from highly cited articles, microglia pyroptosis, exosomes, mesenchymal stem cells, and microglial imaging (PK11195, TSPO, pet imaging) are also receiving more attention, which can compensate for the dearth of clustering research (Delpech et al., 2019; Voet et al., 2019; Kreisl et al., 2020).

Literature is the basis of knowledge structures. We will break this into five sections to make it easier to understand. Firstly, the review articles, which are (Kreutzberg, 1996; Block et al., 2007; Hanisch and Kettenmann, 2007; Ransohoff and Perry, 2009; Kettenmann et al., 2011), can help us to understand the basic research situation and progress of microglia quickly. The second is the monitoring function of microglia. According to Nimmerjahn et al. (2005) and Davalos et al. (2005), microglia can move in a specific direction when the P2Y receptor (especially P2Y12, Haynes et al., 2006) is active. This function of microglia includes monitoring and detecting the microenvironment under physiological or pathological conditions.

Furthermore, Cardona AE 2006 confirmed the critical function of the CX3CR1 receptor in microglia, maintaining the homeostasis of the microenvironment. The third is the origin of microglia. Simard et al. (2006) found that bone marrow-derived microglia could phagocytize plaques in AD. Mildner et al. (2007) found that peripheral monocytes could colonize intracranial microglia only under specific conditions (such as blood–brain barrier injury). Until 2010, Ginhoux et al. (2010) used lineage tracing to confirm that microglia were transformed from yolk sac primordial macrophages on the eighth day of the embryonic stage, which resolved the dispute for many years. Then comes the synaptic pruning function of microglia. In 2009, Wake et al. (2009) found that microglia have a synaptic monitoring function. Paolicelli et al. (2011) confirmed that microglia play an important role in synaptic development. Schafer et al. (2012) and Parkhurst et al. (2013) found that microglia can achieve synaptic pruning function through CR3/C3 and promote synaptic formation by paracrine BDNF. Finally, a recent study, Butovsky et al. (2014), used single-cell sequencing technology to identify specific microglia genes and molecular markers, but these were not expressed in monocytes or other macrophages. Elmore et al. (2014), who used PLX3397 to target microglial CSF1R and deplete microglia, opened a more direct path for the study of microglia function. Keren-Shaul et al. (2017) defines a group of disease-related microglia in neurodegenerative diseases. According to our analysis of the top 30 burst literature (Supplementary Figure S5), the two recent articles, Krasemann et al. (2017) and Hammond et al. (2019), also fall under the use of single-cell sequencing technology to define the distinctive phenotype of microglia in the course of the disease. This direction is more likely to provide in-depth insights into the mechanism of microglial phenotype and disease association and extend to a broader disease spectrum. We also performed a clustering analysis of the co-cited documents to enhance the creation of the knowledge domains using keywords. Only the transcriptional signature/DNA methylation, post-cardiac arrest brain injury/cardiac arrest, and TBI/TBI formed new clusters in the clustering view, where the cited documents formed 17 clusters, the majority of which are related to keyword clustering. It was proposed that the study of microglia and the development of specific disease research increasingly rely on single-cell sequencing (De Vlaminck et al., 2022; Marsh et al., 2022; Sankowski et al., 2022) and epigenetic regulation (Martins-Ferreira et al., 2021).

This paper also has some limitations. First, because this paper only considers literature published between 2002 and 2021, there may be some omissions in the important literature, especially those published within the last 3 years, because the related analysis of the number of citations may be impacted by the publication time; for instance, Wendeln’s 2018 research on immune memory and microglia priming (Wendeln et al., 2018), and Bottcher’s 2019 research on the sequencing of human microglia (Böttcher et al., 2019). Secondly, for accuracy, only the author’s keywords are used in keyword analysis because some articles may not have author keywords, meaning some information may be omitted.

Finally, according to the knowledge domains and dynamic evolution we analyzed, some possible future research directions can also be put forward. Mainly including: 1. Microglial development (Wurm et al., 2021; Iyer et al., 2022) and phenotypes in physiological or pathological conditions, also involving microglial heterogeneity in spatial and temporal (Masuda et al., 2019), gender (Dockman et al., 2022; Fiore et al., 2022), or other dimensions. 2. The critical role of communication/conversation between microglia-neurons (Cserép et al., 2021) and microglia-astrocytes (Han et al., 2021) under ontogeny, physiology, and pathology. 3. Studies on the functional mechanism of microglia by metabolic reprogramming (Minhas et al., 2021), exosomes (Zhu et al., 2022), and epigenetic modification (Martins-Ferreira et al., 2021). 4. In terms of animal models, zebrafish (Silva et al., 2021) may be given more attention. 5. More extensive applications of pet imaging (Kreisl et al., 2020), two-or multi-photon imaging (Scheiblich et al., 2021), and single-cell sequencing (Sankowski et al., 2022) will continue to contribute to the study of microglia. 6. The disease spectrum of microglial study will be broadened, and the popularity of neurodegeneration (Silvin et al., 2022), stroke (Beuker et al., 2022), psychiatric disorders (Fang et al., 2021), and TBI (Luo et al., 2021) research will continue. 7. It is imperative to define, standardize, and unify the nomenclature of microglia (Paolicelli et al., 2022), which will lay a solid foundation for the study of microglia.

## Conclusion

In this study, we explore the study of microglia from 2002 to 2021 using bibliometrics methods in conjunction with CiteSpace and VOSviewer software. The fundamental data, knowledge domains, evolution, and emerging trends were built with the intention of supplying pertinent researchers with concepts to comprehend and delve deeply into the field of microglia research.

## Data availability statement

The original contributions presented in the study are included in the article/Supplementary material, further inquiries can be directed to the corresponding authors.

## Author contributions

GL contributed to the conception and performed the experiment of the study. TL performed the experiment and contributed significantly to the analysis and manuscript preparation. AY performed the data analyses. XZ helped write the manuscript. SQ and WF helped perform the analysis with constructive discussions. All authors contributed to the article and approved the submitted version.

## Funding

This study was supported by grants from The National Nature Science Foundation of China (NSFC): No. 82101398.

## Conflict of interest

The authors declare that the research was conducted in the absence of any commercial or financial relationships that could be construed as a potential conflict of interest.

## Publisher’s note

All claims expressed in this article are solely those of the authors and do not necessarily represent those of their affiliated organizations, or those of the publisher, the editors and the reviewers. Any product that may be evaluated in this article, or claim that may be made by its manufacturer, is not guaranteed or endorsed by the publisher.

## Abbreviations

AD: Alzheimer’s disease
PD: Parkinson’s disease
TBI: traumatic brain injury
ROS: reactive oxygen species
NO: nitric oxide
IL-1β: interleukin-1β
TNF-α: tumor necrosis factor-α
Avg. norm. citations: averaged normalized number of citations
MAPK: mitogen activated protein kinase
C.C.: citation counts
Bur.: burstness intensity
Sig.: sigma value
