# Supplementary figures and images for: Knowledge domains and emerging trends of microglia research from 2002 to 2021: A bibliometric analysis and visualization study
Source: Front Aging Neurosci. 2023 Jan 5;14:1057214. doi: 10.3389/fnagi.2022.1057214 (PMC9849393; doi:10.3389/fnagi.2022.1057214)

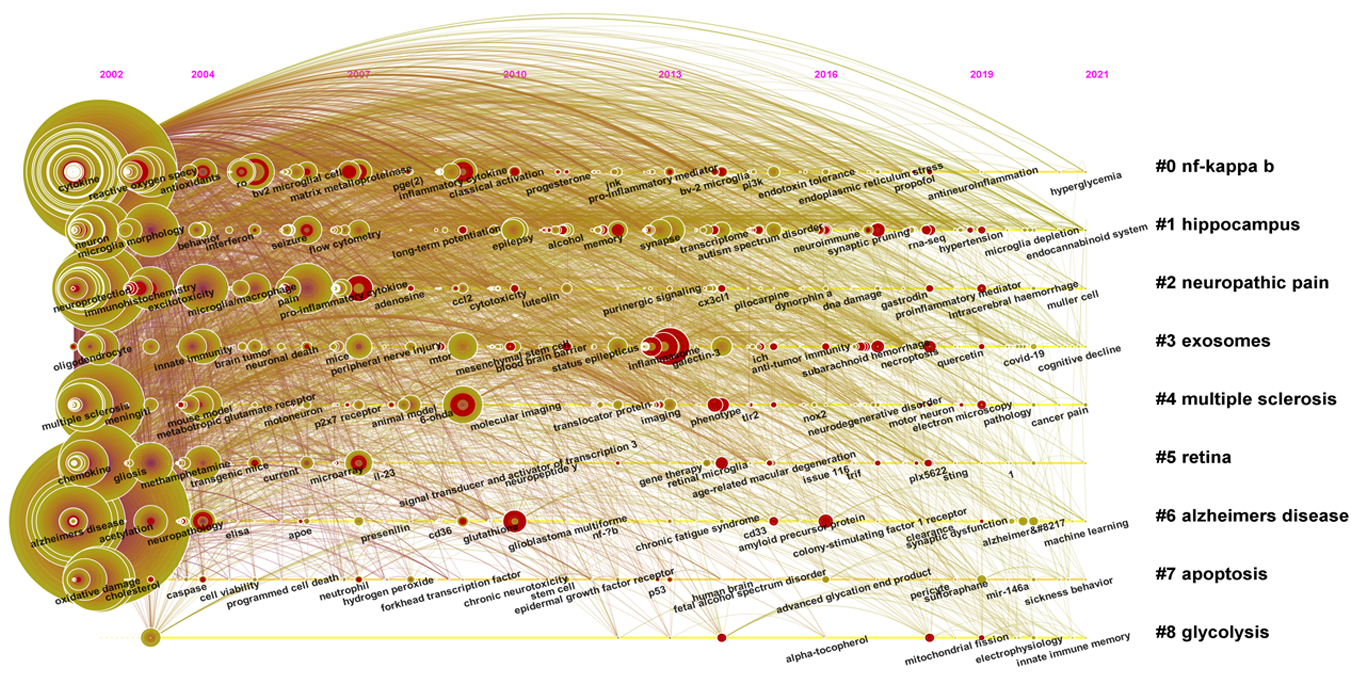

Supplement: Supplementary file 1 [file Image_1.TIF]

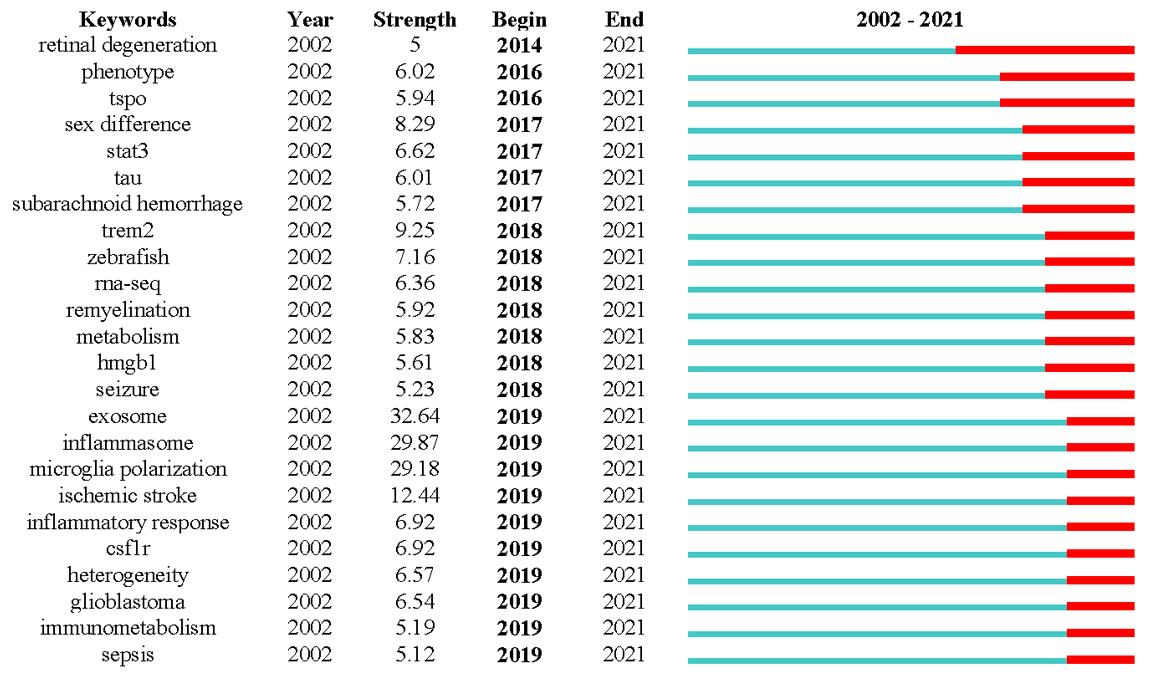

Supplement: Supplementary file 2 [file Image_2.TIF]

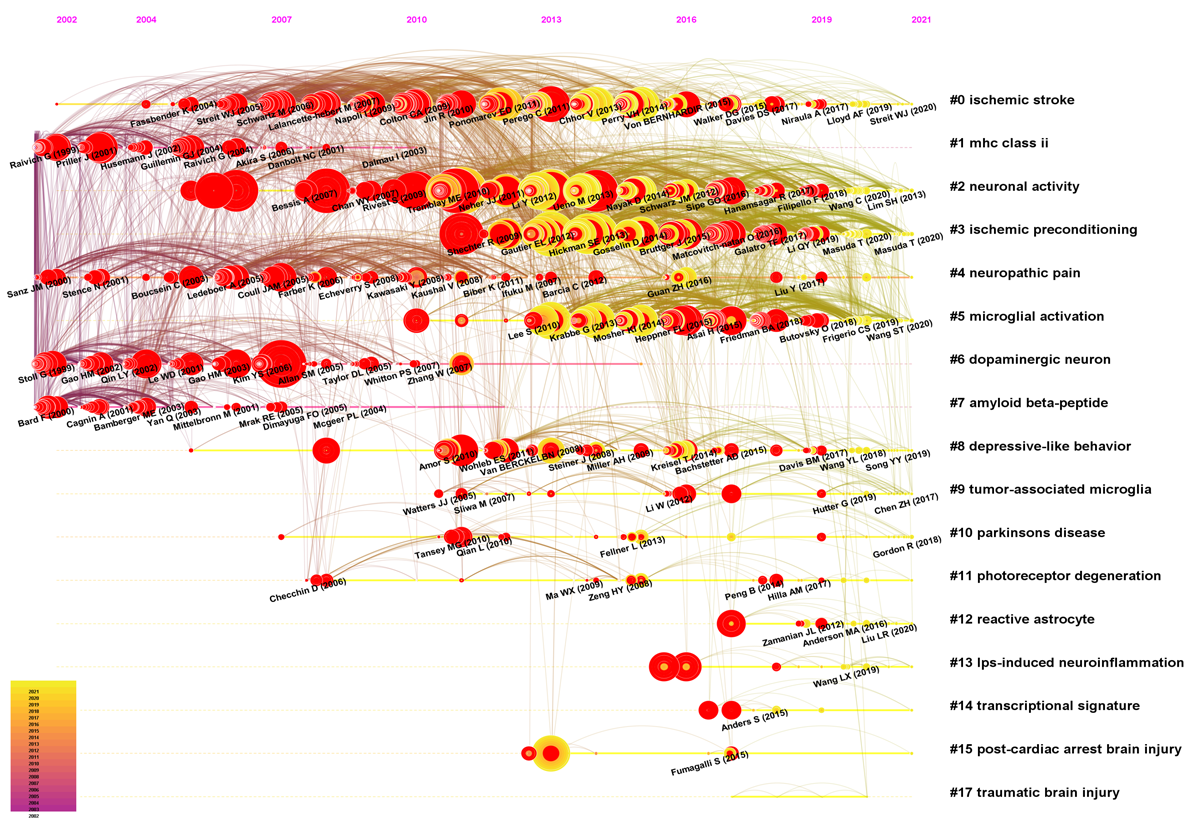

Supplement: Supplementary file 3 [file Image_3.TIF]

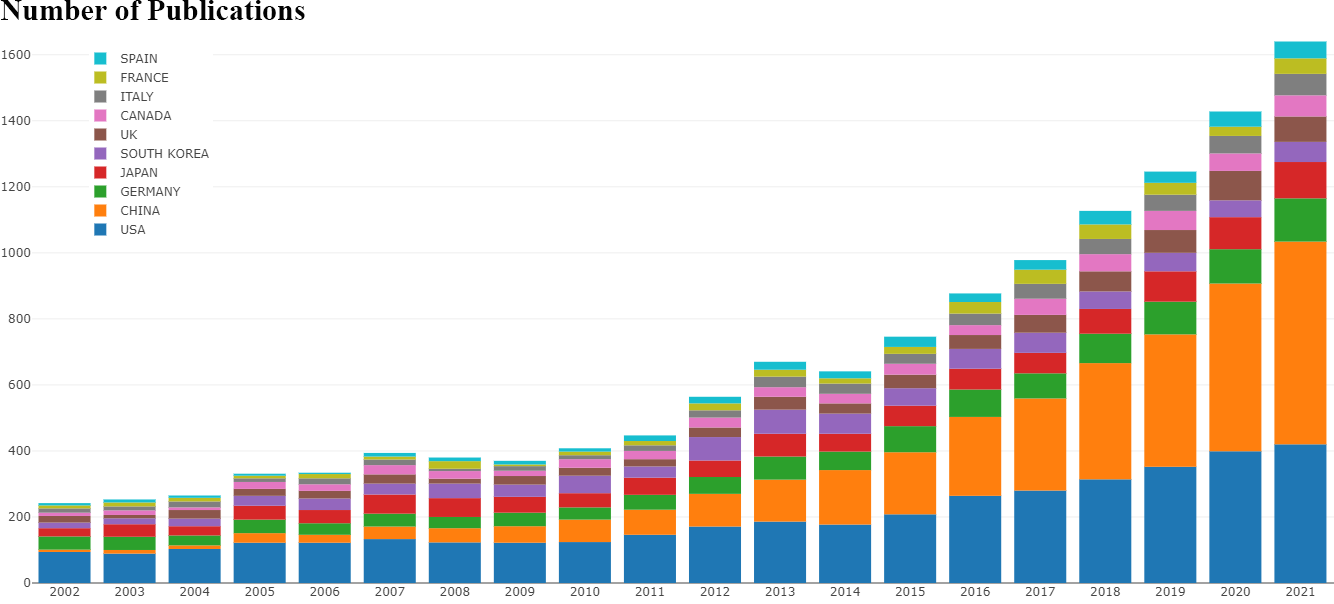

Supplement: Supplementary file 4 [file Image_4.TIF]

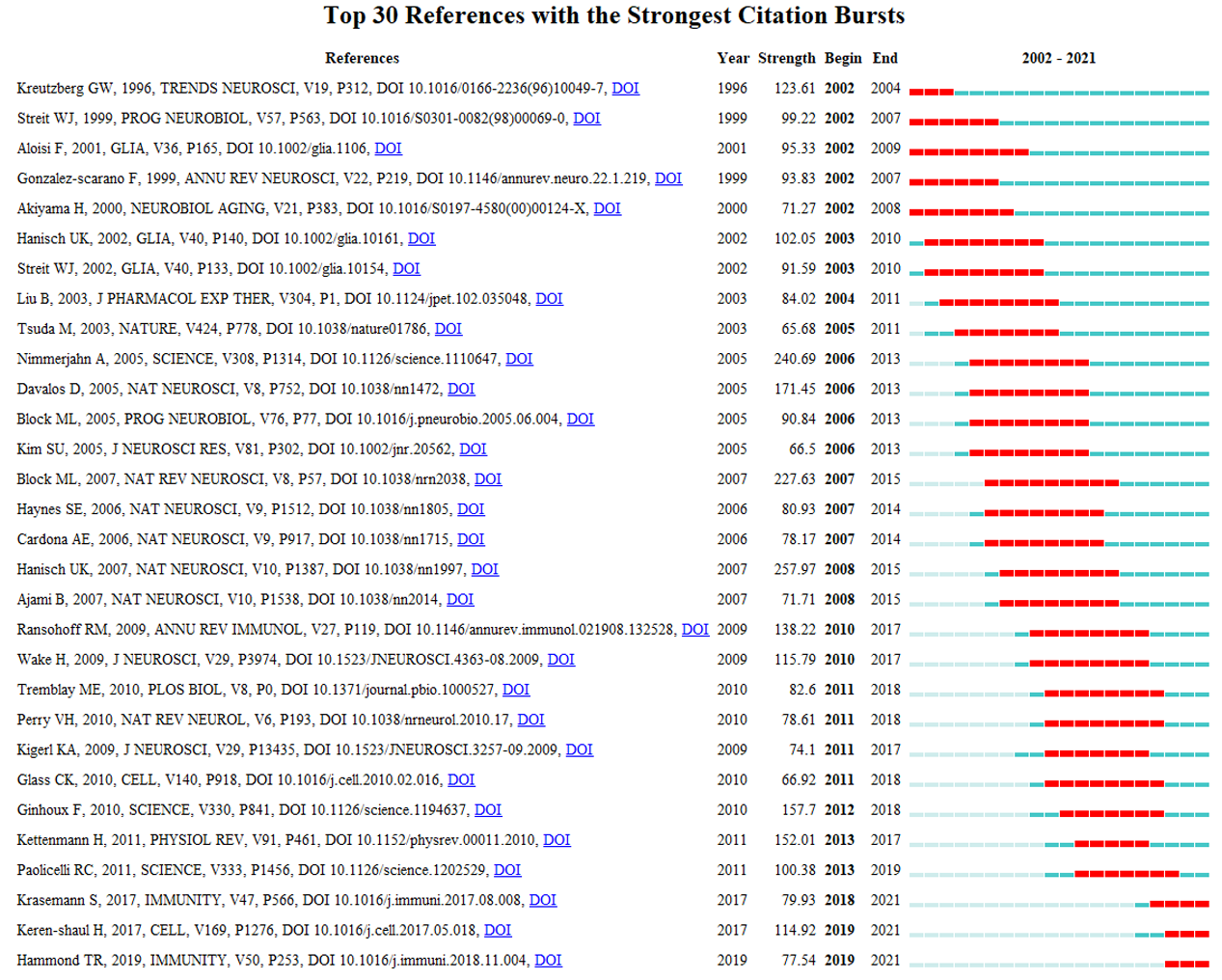

Supplement: Supplementary file 5 [file Image_5.TIF]
