# Supplementary material for: Knowledge domains and emerging trends of microglia research from 2002 to 2021: A bibliometric analysis and visualization study
Source: Front Aging Neurosci. 2023 Jan 5;14:1057214. doi: 10.3389/fnagi.2022.1057214 (PMC9849393; doi:10.3389/fnagi.2022.1057214)
Supplement: Supplementary file 6 [file Table_1.DOCX]

Supplementary Material

**Table S1** The top 10 institutions involved in microglia research field

| Organization | Documents | Centrality | Avg. norm. citations | | Total link strength | |
| --- | --- | --- | --- | --- | --- | --- |
| Harvard Univ | 267(2.08%) | 0.15 | | 2.67 | | 600 |
| Kyushu Univ | 204(1.59%) | 0.07 | | 1.23 | | 237 |
| Shanghai Jiao Tong Univ | 193(1.51%) | 0.04 | | 0.86 | | 198 |
| Fudan Univ | 153(1.19%) | 0.02 | | 1.09 | | 183 |
| Univ Freiburg | 145(1.13%) | 0.04 | | 2.29 | | 311 |
| Chinese Acad Sci | 140(1.09%) | 0.03 | | 1.02 | | 253 |
| Kyung Hee Univ | 138(1.08%) | 0.02 | | 0.59 | | 129 |
| Sun Yat Sen Univ | 137(1.07%) | 0.02 | | 0.91 | | 152 |
| Huazhong Univ Sci & Technol | 136(1.06%) | 0.03 | | 0.79 | | 161 |
| Univ British Columbia | 134(1.05%) | 0.05 | | 1.16 | | 201 |

**Table S2** Summary of the 17 clusters of co-citation networks

| Cluster | Size | Silhouette | Label (Title/ Keywords) | Average Year |
| --- | --- | --- | --- | --- |
| 0 | 316 | 0.762 | ischemic stroke/ stroke | 2012 |
| 1 | 202 | 0.826 | mhc class ii/ chemokine | 2003 |
| 2 | 202 | 0.799 | neuronal activity/ synapse | 2015 |
| 3 | 190 | 0.867 | ischemic preconditioning/ microglial depletion | 2017 |
| 4 | 169 | 0.936 | neuropathic pain/ neuropathic pain | 2009 |
| 5 | 167 | 0.907 | microglial activation/ alzheimer’s disease | 2017 |
| 6 | 164 | 0.887 | dopaminergic neuron/ parkinson’s disease | 2005 |
| 7 | 115 | 0.901 | amyloid beta-peptide/ beta-amyloid | 2004 |
| 8 | 83 | 0.941 | depressive-like behavior/ depression | 2015 |
| 9 | 32 | 0.99 | tumor-associated microglia/ glioma | 2016 |
| 10 | 30 | 0.984 | parkinsons disease/ parkinsons disease | 2016 |
| 11 | 28 | 0.976 | photoreceptor degeneration/ retina | 2014 |
| 12 | 11 | 0.999 | reactive astrocyte/ astrocytes | 2019 |
| 13 | 11 | 0.994 | lps-induced neuroinflammation/ glycolysis | 2019 |
| 14 | 7 | 0.999 | transcriptional signature/ dna mythylation | 2018 |
| 15 | 7 | 0.996 | post-cardiac arrest brain injury/ cardiac arrest | 2016 |
| 17 | 4 | 1 | traumatic brain injury/ traumatic brain injury | 2018 |
